# The Intra-Examiner Variability in and Accuracy of Traditional Manual Diagnostics of Benign Paroxysmal Positional Vertigo: A Prospective Observational Cohort Study

**DOI:** 10.3390/jcm14020434

**Published:** 2025-01-11

**Authors:** Malene Hentze, Dan Dupont Hougaard, Herman Kingma

**Affiliations:** 1 Balance & Dizziness Center, Department of Otorhinolaryngology, Head & Neck Surgery and Audiology, Aalborg University Hospital, 9000 Aalborg, Denmarkhermanuskingma@gmail.com (H.K.); 2 Department of Clinical Medicine, Aalborg University, 9220 Aalborg East, Denmark

**Keywords:** vertigo, vestibular diseases, diagnostics, benign paroxysmal positional vertigo, BPPV, variability, accuracy, Dix–Hallpike test, Supine Roll Test

## Abstract

**Background/Objectives:** Accurate head positioning is essential for diagnostics of benign paroxysmal positional vertigo (BPPV). This study aimed to quantify the head angles and angular velocities during traditional manual BPPV diagnostics in patients with positional vertigo. **Methods**: A prospective, observational cohort study was conducted at a tertiary university hospital outpatient clinic. One trained examiner performed the Supine Roll Test (SRT) and the Dix–Hallpike test (DHT) on 198 adults with positional vertigo. The primary outcomes included head angle variability and accuracy and angular velocity variability. The secondary outcomes examined the relationship between the head angle accuracy and participant-reported limitations. **Results**: The absolute variability for all head angles ranged from ±8.7° to ±11.0°. The yaw axis head angles during the DHT, particularly on the left side, had the highest relative variability (left DHT: coefficient of variance 0.29). Systematic errors included the yaw axis head angles undershooting the target (90°) by 19.7–23.8° during the SRT and the pitch axis head angles undershooting the target (120°) by 7.8–8.7° during the DHT. The left-sided yaw axis in the DHT was undershot by 11.8°, while the right-sided DHT angle was slightly overshot (2.5°). Right-sided yaw axis angles in the SRT and DHT were more accurate than the left-sided ones (right SRT: 19.9°; left SRT: 23.9°; *p* < 0.0001) (right DHT: 7.0°; left DHT: 13.2°; *p* < 0.0001). The regression analysis found no association between the participant-reported limitations and head angle accuracy. **Conclusions**: This study highlights the substantial variability and inaccuracies in head positioning during traditional manual BPPV diagnostics, supporting the relevance of a guidance system to improve BPPV diagnostics. Level of evidence: III. Trial registration: ClinicalTrials.gov identifier: NCT05846711.

## 1. Introduction

Benign paroxysmal positional vertigo (BPPV) is a vestibular disorder characterized by recurrent, brief episodes of vertigo triggered by specific changes in head position relative to the gravity vector [1,2]. BPPV is diagnosed in up to 27% of cases presenting with vertigo, making it the leading cause of peripheral vertigo [3,4,5]. While BPPV may affect individuals at any age, it is particularly prevalent among older adults [4]. However, BPPV might be underdiagnosed, as 9% of those referred to a geriatric clinic (none with a current history of balance disorders) were diagnosed with unrecognized BPPV [6]. BPPV significantly affects quality of life [7], with limitations in daily activities such as interruptions to car driving (24%), social isolation (18%), and sick leave (37%) [4]. BPPV can also lead to an increased risk of falls (78% within 3 months) [6], anxiety, and depression [4,6]. If untreated or misdiagnosed, BPPV can lead to multiple medical consultations (with 39% consulting more than one specialty [4]) and unnecessary examinations or interventions (with 77% undergoing additional laboratory testing) [4,7]. Therefore, an accurate diagnosis of BPPV is highly essential from the perspective of patients and healthcare systems.

No definite consensus regarding the underlying pathophysiology of BPPV exists. The most acceptable theory is that BPPV is attributed to the detachment of otoconia from the macula of the utricle [1]. The otoconia then enter one (or more) of the three paired semicircular canals (SCCs) (most frequently the posterior SCC (48–79%), followed by the lateral SCC (17–46%), and rarely the anterior SCC (1–3%)) [8,9,10], where they interfere with the SCC dynamics. The most common pathophysiology subtype is believed to be canalolithiasis, where the otoconia float freely within the endolymph of the SCC. Head movements aligning the plane of the affected SCC with the gravity vector then cause gravity to pull the otoconia debris downward, which induces endolymph flow. This flow then deflects the cupula, eventually leading to the perception of a head rotation (vertigo) and positional nystagmus through excitation of the affected SCC through modulated activity in the vestibular nerve afferents [11]. Although the head is kept still in this position, the illusion of rotation (vertigo) will last for many seconds until the debris is not moving anymore and the cupula has returned to the rest position. Another, more debated pathophysiology subtype is represented by cupulolithiasis, where the otoconia are believed to settle down onto the cupula of the SCC, increasing its density. Head movements aligning the affected SCC with the gravity vector cause gravity to pull the heavy cupula downward, changing the firing rate of the vestibular nerve and causing vertigo and positional nystagmus. In cupulolithiasis, the vertigo and nystagmus continue for more than one minute (until central adaptation causes them to fade) [12]. However, evidence for the theory of cupulolithiasis in humans is lacking, and cupulolithiasis is believed to be confused with periampullary canalolithiasis and canalith jam [13].

Based on these theories of BPPV’s pathophysiology, BPPV diagnostics rely heavily on clinical examinations, which are traditionally performed manually on an examination bed without any specialized equipment. BPPV diagnostics consist of positional tests that position the patient’s head so that the plane of the investigated SCCs is aligned with the gravity vector. This causes, as indicated above, otoconia and/or cupula movements, leading to positional vertigo and positional nystagmus. The most frequently used diagnostic tests are the Dix–Hallpike test (DHT) for examining the vertical (posterior and anterior) SCCs and the Supine Roll Test (SRT) for examining the horizontal (lateral) SCCs [1,2,14]. As a diagnostic criterion, the type of positional nystagmus induced (horizontal, vertical, and/or torsional) must agree with stimulation of the examined SCC, and the time course of the positional nystagmus should agree with either canalolithiasis or cupulolithiasis [1,2,14]. It is crucial to confirm that the observed nystagmus aligns with the BPPV pattern of the stimulated SCC to avoid overdiagnosing BPPV. Positional nystagmus is a common finding in 71–88% of healthy individuals (without positional vertigo) who undergo positional diagnostic tests [15,16]. However, positional nystagmus in individuals without BPPV can be differentiated from BPPV nystagmus by its characteristics: a low slow-phase velocity, prolonged duration, and the absence of a torsional component [15,16]). To enhance the diagnostic accuracy, an additional diagnostic criterion requires positional vertigo to either be present in the patient’s history [1,14] or to be concomitant with positional nystagmus during the diagnostic tests [2].

Due to their accessibility in clinical practice, traditional manual diagnostic tests are widely performed across different healthcare providers in vestibular medicine, including general practitioners, neurologists, otorhinolaryngologists (ENT), and physiotherapists.

Although it is theoretically simple, performing head positioning precisely presents challenges in manual BPPV diagnostics. The core of this challenge might be a combination of (1) the patient’s ability to cooperate [17,18], (2) imprecise positioning of the head between and within examiners [19,20], and (3) suboptimal velocities during the positioning. These diagnostic challenges can lead to suboptimal and delayed treatment or misdiagnosis, with substantial consequences for patients and healthcare systems.

In general, a sparse amount of literature that directly evaluates the variability in and accuracy of head angles exists, and even fewer examples in the existing literature focus on the angular velocities and duration of movements with the management of BPPV. Some of the most current research that focuses on the treatment of BPPV has reported significant inter-examiner variability [19] and angular misalignment (low accuracy) [21,22] for every head position included in the Epley maneuver. Lieberz et al. [20] examined the same matter for BPPV diagnostics using the DHT. They described a significant difference between the target head angles and those obtained (accuracy), which was more pronounced between examiners (inter-examiner) than when performed by the same examiner (intra-examiner).

To our knowledge, all existing studies on the DHT have used a healthy test population, and none have yet described the variability in and accuracy of the SRT and the DHT in patients with positional vertigo. As many clinical studies are performed on symptomatic patients, it is highly relevant to investigate the variability in and accuracy of these diagnostic tests in this population.

This study’s objective addresses this gap in the knowledge by quantifying the intra-examiner variability in and accuracy of head angle measures, the variability in the angular velocities, and the movement duration in traditional manual BPPV diagnostics (using the SRT and the DHT) in a population with positional vertigo. Additionally, this study aims to explore whether participant-reported limitations influence the intra-examiner accuracy of the applied head angles.

## 2. Materials and Methods

### 2.1. Study Design

This study employed a prospective observational cohort design. The design adhered to the Strengthening the Reporting of Observational Studies in Epidemiology (STROBE) Statement. This study formed part of a more extensive, randomized, controlled study comparing traditional manual diagnostics to diagnostics with a mechanical rotation chair [18]. However, this study focused specifically on the detailed positioning during the traditional manual diagnostics. Consequently, it maintained an observational design independent of the randomized trial component. As a practical consequence of this study’s attachment to a larger study, all of the participants underwent the BPPV diagnostics twice (once with traditional manual diagnostics and once with mechanical rotation chair diagnostics), with the order of the methods randomized. One examiner performed all of the examinations. The examiner had prior experience in performing the traditional manual diagnostics corresponding to a junior resident doctor in the specialty of otorhinolaryngology (ENT). After this, the examiner was trained and supervised by two neurotology experts until the examiner was considered an expert. To ensure consistency of the procedure between the study periods, a follow-up supervision was scheduled at the end of the study period. Ad hoc supervision by the neurotologists was provided when the examiner deemed it necessary. The examiner was blinded to the head angles and angular velocities obtained and received no visual feedback during the traditional manual diagnostics.

### 2.2. Participants and Setting

This study took place between 12 April 2023 and 11 January 2024 at a highly specialized tertiary outpatient clinic (Balance and Dizziness Center, Department of Otorhinolaryngology, Head and Neck Surgery and Audiology, Aalborg University Hospital, Denmark). The participants were referred by general practitioners (North Denmark Region) and private ENT clinics (North and Central Denmark Regions). The examiner assessed eligibility upon a participant’s arrival. The inclusion criteria included an age over 18 years, a typical BPPV case history (short-lasting episodes of positional vertigo triggered by head movements relative to gravity), and sufficient Danish language proficiency to understand informed consent. The exclusion criteria covered insufficient cooperation for manual diagnostics and neck and spine immobility preventing manual diagnostics. Concerning the attachment to the more extensive study, subjects were also excluded in cases of any spontaneous or gaze-evoked nystagmus, physical limitations to the mechanical rotation chair diagnostics (weight ≥ 150 kilos and or height ≥ 2 m), comorbidities (heart failure (ejection fraction < 40%), known cerebral aneurysms, recent cerebrovascular events (<3 months), or arterial dissection disease), pregnancy, or sedative antihistamine intake within the past seven days. This study’s sample size aligned with the power calculation in the larger study. No separate sample size calculation was performed for this descriptive study.

### 2.3. Materials

**Figure 1:**
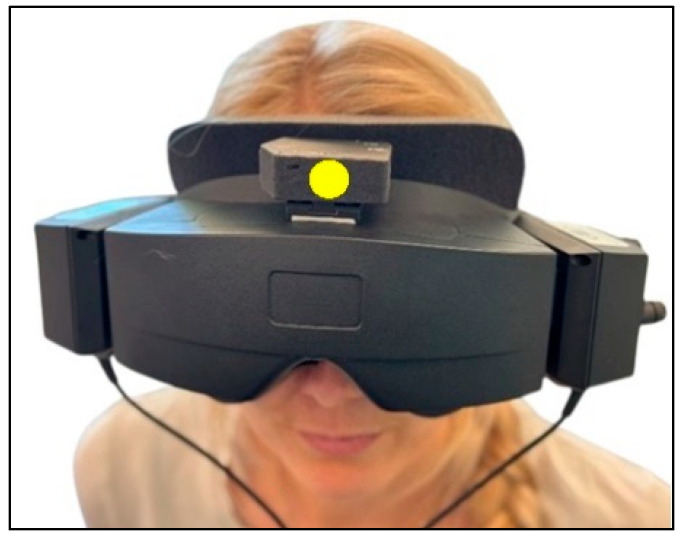
Videonystagmography goggles with inertial measurement unit sensor. All participants were fitted with the videonystagmography goggles with the 6 degrees of freedom inertial measurement unit attached on top of the goggles (marked with a yellow dot).

The participants were fitted with videonystagmography (VNG) goggles (VF405^®^, Interacoustics©, Middelfart, Denmark) equipped with an inertial measurement unit (IMU) sensor (VORTEQ^TM^, Interacoustics©, Middelfart, Denmark) attached (Figure 1). The IMU sensor, incorporating a 3-axis accelerometer and a 3-axis gyroscope, tracked the head positions (static) and movement velocities (dynamic) in 6 degrees of freedom (6DOF). Used during the traditional manual diagnostics and integrated into the software (Micromedical VisualEyes™, version 3.1.0.203, Interacoustics©, Middelfart, Denmark), the 6DOF IMU sensor collected the head position and angular velocity data in real time with a sampling rate of 250 Hz (wirelessly via Bluetooth). When necessary, the 6DOF IMU sensor was connected to the computer via a cable, without affecting the performance of the traditional manual diagnostics (cable sampling rate: 500 Hz).

**Figure 2:**
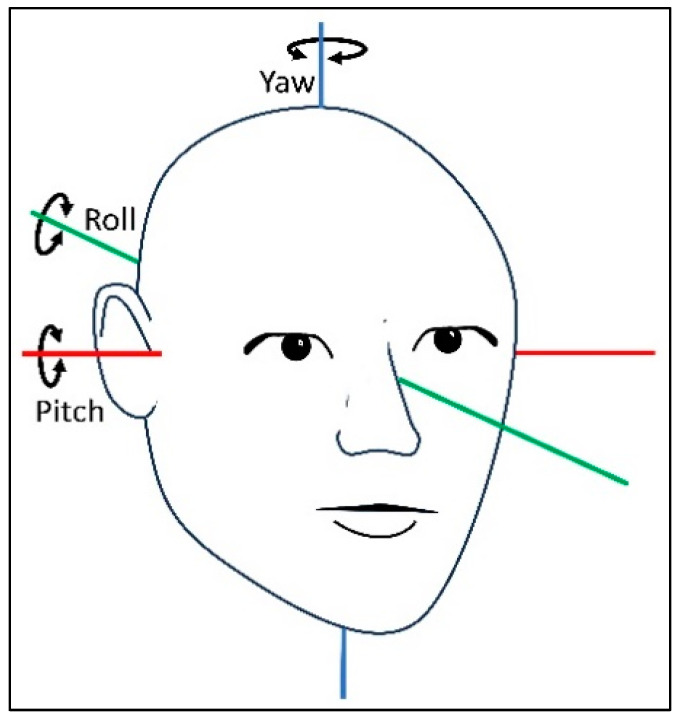
The three axes of rotation describing the orientation of the human head. Movements around the pitch, roll, and yaw axes describe the orientation of the human head.

In collaboration with biomedical engineering students, software was developed to process and display the extracted raw data. The raw data were presented as quaternions divided into vector components (defined by the three dimensions x, y, and z) and a scalar component (defined as the rotation around the dimensions: w). The x, y, and z rotation angles correspond to the pitch, roll, and yaw axes (Figure 2). Initially, in quaternions, the extracted data were transformed into Euler angles and degrees [23]. The software was programmed to define the static and dynamic phases of the diagnostic tests. Static phases represented stationary head positions, while dynamic phases indicated head movements exceeding a 13° threshold within 1 pitch or yaw axis samples from the SRT and the DHT. The software calculated the mean head angles (°) for the static phases. For the dynamic phases, the mean and peak angular velocities (°/second) and total movement duration (second) were calculated. The supine position and the right and left sides in the SRT appeared in the same window, while the DHT displayed each laterality separately. Each test was visualized as a head position–time graph (Supplementary Material Figure S1–S3).

### 2.4. Traditional Manual BPPV Diagnostics

Related to the more extensive study, all of the participants underwent screening for spontaneous and gaze-evoked nystagmus and vestibulo-ocular reflex testing, including a fixation suppression test (manual yaw axis rotation in a mechanical rotation chair with and without fixation). Abnormal findings (less than 70% suppression) led to exclusion, with additional examinations in accordance with the local clinical guidelines.

Before each diagnostic test, the 6DOF IMU sensor was calibrated with the participant seated upright with their head positioned neutrally. The participants were instructed to maintain their head position while the examiner controlled the movements during the diagnostic examination. To prevent displacement of the VNG goggles, and hence the 6DOF IMU sensor, the examiner was careful to maintain a fast grip, with their hands around the headband of the VNG goggles for the entire examination.

**Figure 3:**
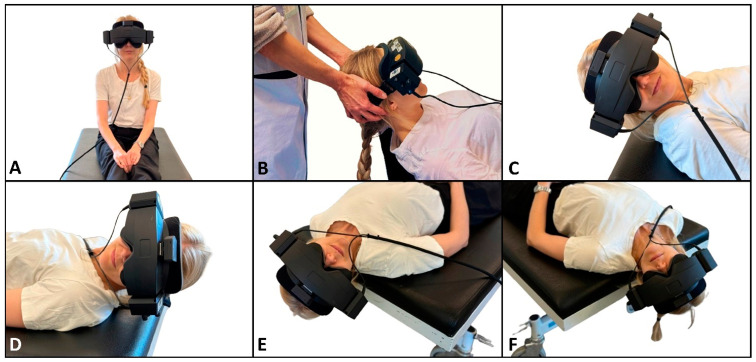
Traditional manual BPPV diagnostics. (**A**) Starting position. The participant sits upright with their head in a neutral position and is fitted with the videonystagmography goggles with an inertial measurement unit sensor attached on top. (**B**) Cranial position of the examiner with the participant in the supine position, with their neck flexed 30° (rotation around the pitch axis by −60° from the starting position). (**C**,**D**) Right and left sides in the Supine Roll Test, respectively. From the supine position, the head is rotated 90° to the right, followed by a 180° rotation to the left (rotation around the yaw axis by ±90°). (**E**,**F**) Right and left sides in the Dix–Hallpike test, respectively. Again, the participant initially sits upright (**A**). Their head is then rotated 45° to the side of the examined posterior canal (the contralateral side of the anterior canal) (rotation around the yaw axis by ±45°) and then moved backward to the supine position with the neck extended to 30° below the horizontal plane (total rotation of the body around the pitch axis by −120°).

The traditional manual diagnostics were performed with the examiner in the cranial position in relation to the participant and followed a standardized order of positions (Figure 3): the upright position, the supine position with the neck flexed (30°), the head rotated (90°) to the right around the yaw axis (right SRT), the head rotated (180°) to the left around the yaw axis (left SRT), the upright position with the head in a neutral position, the head rotated (45°) to the right around the yaw axis and then body positioned supine with the neck extended (30°) (right DHT), the upright position with the head in a neutral position, the head rotated (45°) to the left around the yaw axis and then the body positioned supine with the neck extended (30°) (left DHT), and the upright position with the head in the neutral position. The right and left positions in the SRT were held until positional nystagmus was detected and interpreted. When no nystagmus was observed the SRT positions were held for a maximum of 30 s.. The DHT positions were held for 60 s. We aimed for all changes in position to be completed within 2 s. The target head angles were the ones defined in the Bárány Society diagnostic criteria for BPPV, which are based on the underlying anatomy of the SCCs [1]. Please refer to Figure 3 for a detailed description and visualization of the positioning profile in the traditional manual diagnostics.

The examiner assessed the participants’ cooperation on site, categorizing it as (1) sufficient cooperation (no restrictions), (2) compromised but acceptable (at least 2/3 of the target 90° yaw axis rotation in the SRT and at least 2/3 of the target 45° yaw axis rotation or 100° pitch axis rotation in the DHT), or (3) insufficient (less than 2/3 of the target 90° yaw axis rotation in the SRT and less than 2/3 of the target 45° yaw axis rotation or 100° pitch axis rotation in the DHT). Participants with insufficient cooperation were excluded. The examiner made annotations of any complaints by the participants during the traditional manual diagnostics. The complaints were categorized into four groups: neck and back pain, anxiety, nausea and/or vomiting, and difficulty sitting upright during the diagnostic tests. These four categories were combined into one overall binary variable named participant-reported limitations.

### 2.5. Data Collection

The 6DOF IMU sensor continuously recorded the head angulation and angular velocity throughout the examinations. The raw data were extracted using a research module and translated into the quantitative variables of interest using the custom-developed software. The examiner collected the baseline data through history-taking and reviewing electronic patient records. All variables were entered into REDCap^®^ (Research Electronic Data Capture) version 13.1.37, hosted on a secure server in the North Denmark Region [24,25]. Rigorous data quality checks identified and excluded low-quality data. When the software’s automatic phase detection proved to be inadequate, recalculations were performed.

### 2.6. Statistical Analysis

Descriptive statistics were calculated for the baseline data, head angles, angular velocities, and movement duration for both the SRT and the DHT. Continuous variables were checked for normality visually (with QQ plots and histograms) and using the Kolmogorov–Smirnov test for normality. In cases of a non-normal distribution, bootstrapping was used to calculate the mean and standard deviation (SD). Categorical variables were summarized using absolute and relative frequencies. The chi-square test examined trends between categorical groups (Fisher’s exact test was applied when the expected cell counts were below 5).

The head angles and angular velocities (mean and peak) were reported with the means (including the range, standard deviation (SD), and coefficient of variance (COV)) and the difference between the target and obtained head angles (including the 95% confidence intervals). The variability in the head angles, angular velocities, and movement durations was expressed as the SD (absolute variability) and the COV (relative variability). The accuracy of the head angles was expressed as the absolute difference between the target and obtained head angles. The raw difference between the target and obtained head angles was used to estimate any systematic errors in the performance of the diagnostic tests. For clarity, only the most relevant head angles for the SRT and the DHT were displayed. The accuracy of the angular velocities and movement durations was not calculated, as we did not have any predetermined target values.

A paired Student’s *t*-test was used to compare the intra-examiner accuracy between the test laterality and the two diagnostic tests. Bootstrapping was employed for non-normally distributed data. To test the relationship between the participant-reported limitations and the accuracy of the head angles, a multiple regression analysis adjusted for age was employed. The model was built based on expert knowledge, constructing a directed acyclic graph based on the data variables collected. Before each regression model was fitted, a scatterplot visualized the data. All models underwent a post-estimation check, including a scatterplot of the residuals and the fitted values, a scatterplot of the residuals and each independent variable, a QQ plot of the residuals, and a Kolmogorov–Smirnov test for the normality of the residuals. Regression models without normally distributed residuals were fitted with bootstrapping. Potential interaction between age and the variable of participant-reported limitations was also checked.

Post hoc analyses using the unpaired Student’s *t*-test were performed to compare the means of the head angle accuracy between the groups with and without reported limitations during the SRT and the DHT. If the assumption of equal variance was violated, a Welch’s *t*-test was performed. For variables that were not normally distributed, bootstrapping was used in the reported results. To avoid Type I errors, all significant results in these post hoc analyses were Bonferroni-corrected.

All analyses were based on complete datasets, excluding data points of insufficient quality. The selected analyses were chosen in collaboration with an independent certified biostatistician who also provided advice throughout the study period. The results were evaluated with significance at the alpha level of 0.05. All data processing and analyses were performed using Stata/MP 18.0.

## 3. Results

**Figure 4:**
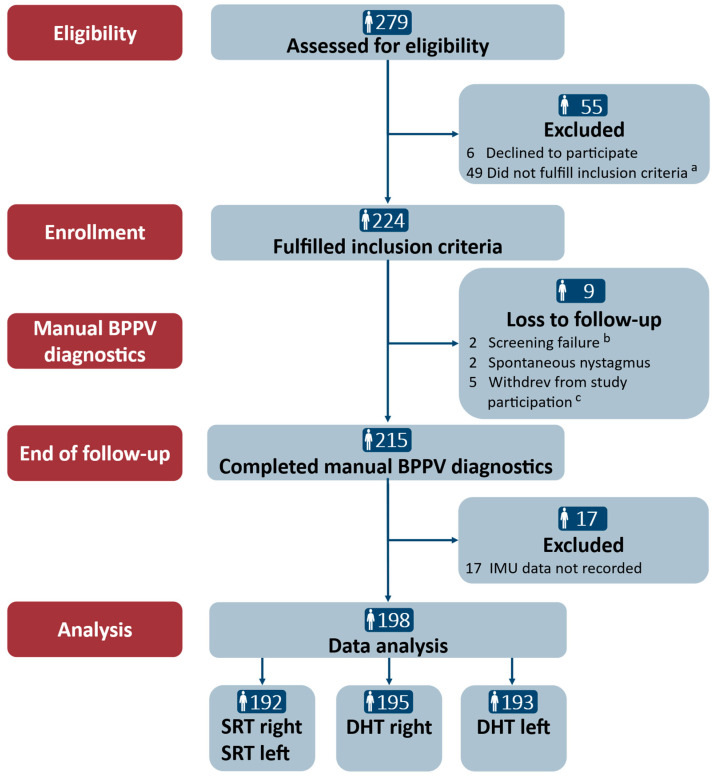
Trial profile. SRT: Supine Roll Test; DHT: Dix–Hallpike test. ^a^ The reasons for the inclusion criteria not being fulfilled in the main study were the remission of vertiginous symptoms (*n* = 34), a lack of understanding of Danish (*n* = 2), neck and back immobility to a degree hindering manual diagnostics (*n* = 5), spontaneous and gaze-evoked nystagmus (*n* = 1), pregnancy (*n* = 1), intake of sedative antihistamines (*n* = 2), and cardiovascular comorbidities (*n* = 4). ^b^ Screening failure included the intake of sedative antihistamines (*n* = 1) and cerebral hemorrhage (<3 months) (*n* = 1). ^c^ Participants withdrew during the study period due to side effects related to the use of the mechanical rotation chair: vomiting (*n* = 1), anxiety (*n* = 2), and claustrophobia (*n* = 2).

Of the 279 participants who were assessed for eligibility, 224 (80.3%) were included. During the study period, nine (4.0%) participants were lost to follow-up: four were excluded, and five withdrew from study participation due to side effects related to the diagnostics with the mechanical rotation chair in the major study (the relationship between this study and the major study is described in the “Materials and Methods” section). Of the 215 participants who completed the study, 17 (7.9%) participants were excluded due to data loss during the Bluetooth transfer from the 6DOF IMU sensor. Hence, a total of 198 participants were included in the data analyses. The data were assessed to be of sufficient quality in the vast majority of the SRTs (*n* = 192) and the DHTs (right: *n* = 195, left: *n* = 192). The examiner and the participants reported no adverse events during the traditional manual diagnostics. Please refer to Figure 4 for a detailed description of the participant flow.

Table 1 shows the baseline characteristics. The population mainly consisted of women (70.7%), with a mean age of 58.6 years (ranging from 18 to 91 years). The majority of the participants were referred by a general practitioner (86.4%) (private ENT practice referrals: 13.6%). Despite the fact that all of the participants presented with a classic BPPV case history, only 39.9% had BPPV when confirmed using traditional manual diagnostics. Twenty-one (10.6%) participants reported limitations during the traditional manual diagnostics, with neck and back pain as the most frequent complaint (*n* = 14). The examiner reported compromised participant cooperation in almost one-fifth (18.7%) of all participants.

**Figure 5:**
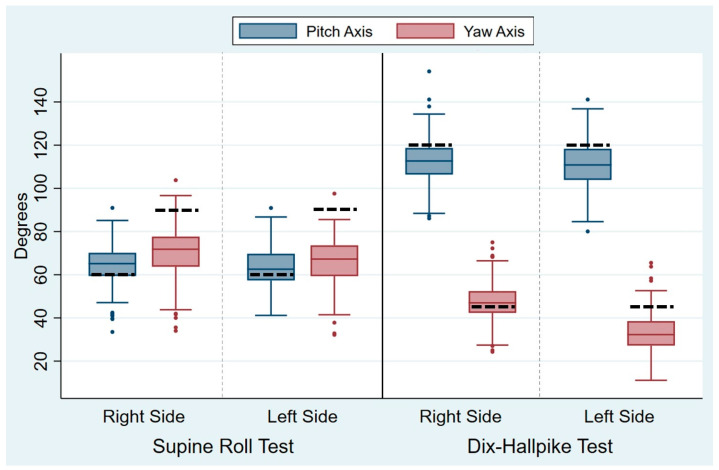
Absolute means of the head angles during traditional manual diagnostics. For clarity of the graphs, the absolute means of the head angles are displayed. Each set of blue and red boxes represents the right and left sides in the Supine Roll Tests (left side of the figure) and the right and left sides in the Dix–Hallpike tests (right side of the figure) for the absolute means of the pitch axis head angles (blue boxes) and the yaw axis head angles (red boxes). The boxes visualize the median quartile (central line through the box), the upper quartile (top of the box), and the lower quartile (bottom of the box). The T-shaped whiskers extend to the maximum (upper) and minimum (lower) data point within 1.5 times the interquartile range. Values further away are considered outliers (dots). The dashed black line represents the target head angles (Supine Roll Test: target pitch angle = 60°; target yaw angle = 90°. Dix–Hallpike test: target pitch angle = 120°; target yaw angle = 45°).

**Figure 6:**
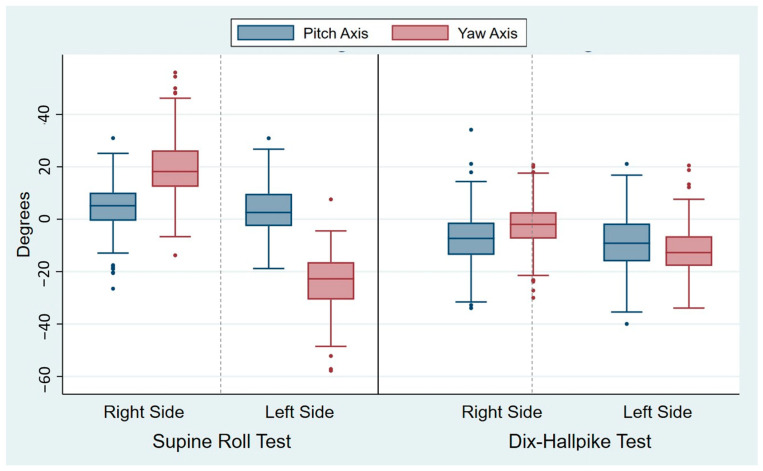
Differences between the target and obtained head angles during traditional manual diagnostics. Each set of blue and red boxes represents the right and left sides in the Supine Roll Tests (left side of the figure) and the right and left sides in the Dix–Hallpike tests (right side of the figure) with the differences between the target and obtained pitch axis head angles (blue boxes) and the target and obtained yaw axis head angles (red boxes). The boxes visualize the median quartile (central line through the box), the upper quartile (top of the box), and the lower quartile (bottom of the box). The T-shaped whiskers extend to the maximum (upper) and minimum (lower) data point within 1.5 times the interquartile range. Values further away are considered outliers (dots). Please note that the most pronounced differences between the target and obtained head angles are seen bilaterally with the Supine Roll Test yaw axis head angles.

The head angles obtained during the traditional manual diagnostics are displayed in Table 2. For all head angles with the SRT and the DHT, there was notable intra-examiner variability (expressed by the SD), which is also visualized by the width of the box plots of the absolute mean of the head angles obtained (Figure 5). The variability was most pronounced for the yaw axis (SD: 10.7–11.0°) of the SRTs and the pitch axis (SD: 10.6–10.9°) of the DHTs. However, the relative variability (expressed by the COV) was greatest for the yaw axis head angles with the DHTs (right COV: 0.19; left COV: 0.29). For all head angles, the difference between the target and obtained head angles was significantly different from 0, meaning that all head angles had significant intra-examiner inaccuracy in relation to obtaining the target head angles. Please refer to Figure 6 for a visualization of the difference between the target and obtained head angles.

When comparing the intra-examiner accuracy between the right and left sides within the same diagnostic test, both the SRT and the DHT displayed a significantly lower intra-examiner accuracy (*p* < 0.0001) for the yaw axis head angles on the left side compared to the right side (Table 3). Also, there was a significant difference between the accuracy of the same head angle axis when compared between the SRT and the DHT: the yaw axis head angles were more accurate in the DHT (*p* < 0.0001), and the pitch axis head angles were more accurate in the SRT (*p* < 0.001) (Table 3).

Table 4 shows the accuracy of the head angles in the groups with and without reported limitations during the traditional manual diagnostics. When compared to the group with no reported limitations, there was a significantly lower accuracy for the yaw axis head angle on the left side in the SRT in both groups with reported limitations compared to the group without (*p* < 0.001). The same pattern applied to the yaw axis head angle on the right side in the SRT in the group with examiner-reported compromised cooperation (*p* < 0.001). For all the remaining head angles during the SRT and the DHT, there were no significant differences between the groups with and without reported limitations during the traditional manual diagnostics. The weak relationship between the accuracy of the head angles and the participant-reported limitations was confirmed by the multiple regression analysis adjusted for age (Table 5). The coefficient slopes indicate no consistent pattern of direction and are non-significant for most of the head angles in the SRT and the DHT (except the yaw axis head angle for the left side in the SRT). The low values of the adjusted R2s also highlight that the variable participant-reported limitations explain only a minor part (or none) of the intra-examiner accuracy in the head angles during the traditional manual diagnostics.

The intra-examiner variabilities in the angular velocities and durations of movements were also notable, with all measures exhibiting a broad range (angular velocity: 11.4–119.7°/s; peak angular velocity: 37.9–353.6°/s; movement duration: 1.0–6.7 s) (Table 6). In the SRT, movement from the right to the left side seems to be performed both at greater velocity and with lower variability (SD ± 9.7°/s) than movement from the supine position to the right side (SD ± 13.8°/s). Using the DHT, there was also a difference between the right and left sides, with the left side having a lower peak angular velocity (121.9°/s) than the right side in the DHT (133.4°/s). The relative variability in the duration of movement from sitting to the supine position was also higher on the left side in the DHT as compared to the right side (right COV: 0.19; left COV: 0.24).

## 4. Discussion

### 4.1. Key Results

This prospective observational cohort study utilized a 6DOF IMU sensor to quantify the head angulations and angular velocities during the traditional manual diagnostics performed by one examiner on the referred participants with a classic BPPV case history.

Variability in the head angles: Our findings confirmed that all the head angles obtained during the SRT and the DHT showed substantial intra-examiner variability (Table 2). Interestingly, for all head angles, the absolute variability was close to ±10°. However, when comparing the relative variability (SD/mean), it was notably higher for the yaw axis head angles in the DHT, particularly on the left side (COV 0.29). These results might indicate that an examiner, regardless of the target angle, will consistently have the same amount of uncertainty attached to the eye measure.

Accuracy of the head angles: Analysis of the systematic errors during the examination (the mean difference between the target and obtained head angles) revealed that the yaw axis head angles during the SRT were consistently undershot by approximately 20° relative to the target angle of 90°. This suggests that achieving the target angle might require not only head movements but also movement of the patient’s entire body. Similarly, for the DHT, the pitch axis head angles were undershot by approximately 10° relative to the target angle of 120°. The same applied to the yaw axis head angle on the left side in the DHT, which was also undershot by about 10°. The yaw axis head angle on the right DHT was the only overshot head angle, although this was minimal (2.5°). Significant inaccuracy was observed for all of the head angles during the SRT and the DHT (Table 2). However, for the yaw axis head angle, the right-sided SRT and DHT were significantly (*p* < 0.0001) more accurate (right SRT: 19.9°; right DHT: 7.0°) than the left-sided counterparts (left SRT: 23.9°; left DHT: 12.2°). However, this difference in laterality did not apply to the pitch axis head angles, where there was no significant difference between the right and left sides in the SRT and the DHT (Table 3). It is important to interpret these significant results with caution. The large sample size reduced the widths of the confidential intervals, potentially leading to statistically significant findings for small group differences that may lack clinical relevance.

When comparing participants with and without reported limitations during the traditional manual diagnostics, we found no significant difference in accuracy for most of the head angles, except for the yaw axis head angles during the SRT (Table 4). However, these post hoc analyses were likely underpowered due to the small subgroup sizes, limiting the certainty of making any conclusions based on them.

A multiple regression model adjusted for age further explored the relationship between the participant-reported limitations and the accuracy of the head angles. The analysis demonstrated that subjective complaints were a weak predictor of intra-examiner (in)accuracy during the traditional manual diagnostics (Table 5). The bad fit of the regression model could be attributed to the limited number of participants reporting limitations (*n* = 21) despite the large overall sample size or the influence of unmeasured confounders. These disregarded variables may have affected both the participant-reported limitations and the accuracy of the head angles, introducing potential bias.

### 4.2. Comparison with the Existing Literature

To our knowledge, this is the first study quantifying the intra-examiner variability in and the accuracy of head angles and the variability in angular velocity when using both the SRT and the DHT in a referred population with positional vertigo. Previous research by Lieberz et al. [20] evaluated the inter- and intra-examiner performance for the right side using the DHT, reporting variability (mean and standard deviation) and a difference between the target and obtained head angles. However, two study design key differences must be considered when comparing these findings: (1) Lieberz et al. reported the variability and accuracy based on the combined inter- and intra-examiner performance, whereas this study focused solely on the intra-examiner performance, and (2) Lieberz et al. aimed for a target pitch axis head angle of −110°, while our study targeted −120°. Similar to our findings, Lieberz et al. reported significant differences between the target and obtained head angles. However, their mean pitch axis head angle overshot the target (−122.6°, SD ± 15.8°), while ours undershot it (−112.3°, SD ± 10.9). Notably, the intra-examiner variability in our study is lower than that reported by Lieberz et al., aligning with their conclusion that inter-examiner variability accounts for the majority (83.4%) of the total variation, with only 16.4% attributed to intra-examiner variation.

Other studies have quantified the head angles during the manual Epley maneuver, used for the treatment of posterior SCC BPPV [19,21,22]. The second step in the Epley maneuver corresponds to the head positions in the supine position used in the DHT. The variability in the pitch axis head angle in this position ranges from 11.4 to 20.0° (lower variability was observed when combining the inter- and intra-examiner performance [21], compared to pure inter-examiner performance [19]). For the yaw axis head angle, the variability ranges from 9.9 to 14.3° [21]. Compared to our intra-examiner variability in the corresponding DHT position (pitch axis: 10.6–10.9°; yaw axis: 9.0–9.6°), the variability is greater between examiners (inter-examiner) than within an examiner (intra-examiner). This observation is trivial and consistent with Lieberz et al.’s findings [20].

Both intra- and inter-examiner differences in accuracy have been reported to be significant in previous studies examining the DHT [20] and the corresponding Epley maneuver positions [21,22]. The accuracy of the yaw axis head angles during the Epley maneuver has been hypothesized to depend on the examiner’s hand dominance, with greater accuracy on the side opposite to the dominant hand [21]. However, the present study did not reproduce this finding. Instead, the right-handed examiner in our study showed lower accuracy on the left side (opposite to the dominating hand) for the yaw axis head angles during both the SRT and the DHT. It could be that these results depend on the position of the examiner, but this has not been studied yet. In summary, the reason for this contrary finding remains unclear.

BPPV diagnostics can potentially be improved using guidance systems to assist examiners in achieving the target head angles, thereby increasing the reproducibility of the head positioning. In relation to the treatment of BPPV (with the Epley maneuver), visual guidance has been shown to reduce the variability significantly [19] and increase the accuracy [22] of the head angles. Similarly, a recent pilot study demonstrated that auditory guidance improved the head angle accuracy during self-administered Epley and Barbeque Roll maneuvers [26]. Given the shared reliance on precise head positioning in BPPV diagnostics and treatment, it is likely that guidance systems could similarly improve the diagnostic accuracy and reduce the variability with traditional manual diagnostics. It is as of yet still unclear how the diagnostic power depends on the accuracy of the head positioning, which is especially difficult to examine, as the reproducibility of the outcome is limited [27].

Limited data regarding the dynamics of the DHT are available. Lieberz et al. [20] reported a mean velocity of 126°/s (SD ± 6.8°/s) for the right side in the DHT, decreasing to 117°/s when the examiner was positioned cranially to the participant. They also reported a mean movement duration of 1.8 s. These values differ substantially from our findings (a mean angular velocity in the right-sided DHT: 46.3°/s, SD ± 9.7°/s; mean movement duration: 2.08 s). This discrepancy likely arose from the differences between study populations. Lieberz et al. [20] examined one single healthy subject trained to undergo the DHT, whereas our study involved “inexperienced” adult participants with positional vertigo. Such differences highlight the importance of considering the population characteristics when interpreting and translating findings into clinical practice.

Another variable that also complicates direct comparison of the results between these studies is the examiner expertise across studies, which ranges from healthcare students or professionals in an undefined specialty [20] and junior ENT doctors [22] to trained BPPV specialists [19,21].

The clinical importance of the variability in and inaccuracy of the head positioning during BPPV diagnostics remains unknown. To our knowledge, no studies have yet investigated the influence of the head positions obtained on diagnostic outcomes. However, it can be hypothesized that diagnostic inaccuracy leads to greater uncertainty in the diagnostic outcome, potentially increasing the risk of misdiagnoses by overlooking actual cases of BPPV. Misdiagnoses might increase the period with symptoms and the risk of a patient undergoing unnecessary additional laboratory testing, hence increasing the burden and socioeconomic healthcare costs.

On the contrary, it might also be that variability in and inaccuracy of BPPV diagnostics are actually an advantage in a heterogeneous population with anatomic variance in the orientation of the SCCs. One previous study found significantly different orientations of the vertical (posterior and anterior) SCCs compared to the assumed anatomic orientation in 83% (5/6) of the angles between the vertical SCCs and the sagittal plane in patients with treatment-resistant BPPV [28].

### 4.3. Strengths and Limitations

A major strength of this study is its large, heterogeneous population, which includes participants of all ages with a typical BPPV case history, enhancing the generalizability of the findings. However, this diversity also introduces a limitation, as it may increase the variability and reduce the accuracy compared to those in studies using healthy test subjects with prior knowledge of traditional manual diagnostics.

In this study, we opted for the consistent use of one examiner, whose training and supervision were carried out by otoneurology specialists to ensure a standardized approach. This allowed us to analyze the variability in executing the diagnostic tests among patients with positional vertigo. However, the major limitation here is that the results also depended on the skills of this single examiner. The examiner performed a high volume of traditional manual diagnostics over a short time, raising the possibility of a learning curve effect influencing the performance of the SRT and the DHT. However, no pattern indicating such an effect was found (Supplementary Material Table S1). In only evaluating one examiner, the size of the inter-examiner variation in a population with positional vertigo remains unknown. However, when comparing this with previous studies, it is reasonable to assume that this variation is larger than the intra-examiner variation. We still recommend that additional studies evaluate inter-examiner variability.

The study population was also influenced by its integration into a larger randomized controlled trial, which necessitated excluding or withdrawing certain participants who might have cooperated with the traditional manual diagnostics alone. Nevertheless, this study’s large sample size and the lack of a selection bias regarding age and sex (Supplementary Material Table S2) mitigate concerns about critical impacts on the data and outcomes.

Measurement uncertainties present another limitation. The 6DOF IMU sensor used was attached to the VNG goggles (Figure 1), capturing the goggles’ orientation rather than the orientation of the head. Efforts were made to minimize displacement through careful and manual stabilization of the goggles during the diagnostics, and visualized data on the revealed movement artifacts were excluded. Ideally, the 6DOF IMU sensor should be fixated onto the skull using methods such as biteboards.

### 4.4. The Impact of the Results and Future Research

The findings in this study are applicable to adult populations undergoing traditional manual diagnostics. Since the results reflect the performance of one examiner, they are inherently dependent on the skill level of this examiner. In this study, the examiner was trained and supervised by two neurotology specialists and had prior experience comparable to that of a junior ENT resident. This reduces the effect of the examiner on the observed variability in and accuracy of manual diagnostics in BPPV patients. The findings provide valuable insights into potential pitfalls in the performance of traditional manual diagnostics, which could enhance diagnostic accuracy, improve patient outcomes, and reduce the burden of BPPV-related morbidity.

Improving the head angle accuracy is likely to enhance the diagnostic precision. Previous research has highlighted the benefits of guidance assistance during BPPV treatment maneuvers, suggesting this approach could also improve diagnostics. As we still do not know the influence of head angle (in)accuracy during traditional manual diagnostics on the diagnostic outcome, we highly recommend future studies investigate the relationship between the head angle accuracy during traditional manual diagnostics and the diagnostic outcomes, with the aim of defining acceptable tolerance windows for the head angles. Additionally, studies exploring the impact of the angular velocities and movement durations on the diagnostic performance could inform more precise clinical guidelines.

To evaluate the performance of traditional manual diagnostics further, test–retest studies assessing their reliability would be highly valuable. The ideal study for evaluating the variability in and accuracy and reliability of traditional manual BPPV diagnostics would be designed to include multiple examiners performing the tests on multiple patients with positional vertigo, providing an in-depth understanding of all three performance measures both within the same examiner (intra-examiner) and between examiners (inter-examiner).

Although this study found a minimal impact of the participant-related variables on the head angle accuracy, it seems unlikely that the examiner’s skills are the sole determinant of the variability in and accuracy of the manual BPPV diagnostics. Therefore, future research should explore potential patient-related factors that might influence the performance of traditional manual diagnostics.

## 5. Conclusions

This study shows that despite these tests being carried out by a trained examiner, there is substantial variability in and inaccuracy of the head angles, angular velocities, and movement durations during the SRT and the DHT in BPPV diagnostics in patients with positional vertigo.

This study found that participant-reported limitations did not clearly influence the head angle accuracy during the diagnostic tests. However, we encourage future studies to explore this matter further as from a clinical perspective, it still seems likely that a participant’s ability to cooperate is crucial to the performance of the diagnostic tests.

The significant inaccuracy in the head positioning emphasizes the need for advanced techniques, such as a guidance system, to improve traditional manual BPPV diagnostics in terms of their reliability, variability, and accuracy. However, it is still unclear how relevant precise head positioning really is to the diagnostic outcome.

Future research investigating the influence of the head positioning accuracy on the diagnostic outcomes and the impact of the use of guidance systems in BPPV diagnostics is encouraged.

## Figures and Tables

**Table 1:** Baseline characteristics (*n* = 198).

Age, Mean ± SD	58.6	15.7
Sex, female, *n* (%)	140	(70.7)
Referred by		
General practitioner, *n* (%)	171	(86.4)
ENT private practice, *n* (%)	27	(13.6)
BPPV diagnosed, yes, *n* (%)	79	(39.9)
Participant-reported limitations		
Participants with self-reported limitations, *n* (%)	21	(10.6)
Neck and back pain during diagnostics, *n* (%)	14	(7.1)
Anxiety during diagnostics, *n* (%)	3	(1.5)
Nausea and/or vomiting during diagnostics, *n* (%)	1	(0.5)
Difficulty sitting upright during diagnostics, *n* (%)	3	(1.5)
Examiner-reported participant cooperation		
Sufficient cooperation ^a^, *n* (%)	161	(81.3)
Compromised cooperation ^b^, *n* (%)	37	(18.7)

ENT: otorhinolaryngologist. ^a^ Sufficient cooperation: the examiner assessed that the Supine Roll Test and the Dix–Hallpike test were conducted without notable deteriorations concerning the target head angle. ^b^ Compromised cooperation: the examiner assessed the Supine Roll Test with head rotations around the yaw axis of a minimum of 2/3 of the target 90° and/or the Dix–Hallpike test with head rotations around the yaw axis of a minimum of 2/3 of the target 45° and/or head rotations around the pitch axis of a minimum of 100°.

**Table 2:** Head positioning in manual BPPV diagnostics (*n* = 198).

Target Head Angle		Obtained Head Angle			Difference Between Target and Obtained Head Angle		Absolute Difference Between Target and Obtained Head Angle		
		Mean [Range]	SD	COV	Mean	95% CI	Mean	95% CI	*p*-Value
Supine Roll Test (*n* = 192)									
Right side									
Pitch axis, °	−60.0	−64.4 [−90.9, −33.5]	±9.3	0.14	4.4	(3.1, 5.8)	8.2	(7.3, 9.0)	0.00 ***
Yaw axis, °	90.0	70.3 [34.0, 103.8]	±11.0	0.16	19.7	(18.1, 21.3)	19.9	(18.4, 21.4)	0.00 ***
Left side									
Pitch axis, °	−60.0	−63.4 [−90.9, −41.2]	±8.7	0.14	3.4	(2.2, 4.7)	7.4	(6.6, 8.3)	0.00 ***
Yaw axis, °	−90.0	−66.2 [−97.6, −32.2]	±10.7	0.16	−23.8	(−25.4, −22.3)	23.9	(22.4, 25.4)	0.00 ***
Right Dix–Hallpike Test (*n* = 195)									
Pitch axis, °	−120.0	−112.3 [−154.1, −86.1]	±10.9	0.10	−7.8	(−9.3, −6.2)	10.6	(9.4, 11.7)	0.00 ***
Yaw axis, °	45.0	47.5 [24.3, 75.0]	±9.0	0.19	−2.5	(−3.7, −1.2)	7.0	(6.2, 7.8)	0.00 ***
Left Dix–Hallpike Test (*n* = 193)									
Pitch axis, °	−120.0	−111.3 [−141.1, −80.1]	±10.6	0.10	−8.7	(−10.2, −7.2)	11.3	(10.2, 12.4)	0.00 ***
Yaw axis, °	−45.0	−33.3 [−65.5, −11.1]	±9.6	0.29	−11.8	(−13.1, −10.4)	13.2	(12.2, 14.3)	0.00 ***

SD: standard deviation, COV: coefficient of variance, CI: confidence interval. All *p*-values are obtained from one sample *t*-test. A *p*-value is considered with a higher level of significance if *p* < 0.0001 (***). Please note that all obtained head angles were significantly different from the target head angles. For the right and left sides in the Supine Roll Test, the yaw axis head angle was undershot. For the Dix–Hallpike test, the most pronounced difference was seen in the yaw axis head angle for the left side in the Dix–Hallpike test, where the head angle was undershot. On the contrary, the yaw axis head angle for the right side in the Dix–Hallpike test was slightly overshot.

**Table 3:** Head positioning accuracy in manual BPPV diagnostics: test laterality (*n* = 198).

	Absolute Differences Between Target and Obtained Head Angles				
	Mean	95% CI	Mean	95% CI	*p*-Value
Comparison of the Right and Left Sides of the Diagnostic Tests					
Supine Roll Test (*n* = 192)	Right Side		Left Side		
Pitch axis, °	8.2	(7.3, 9.0)	7.4	(6.6, 8.3)	0.09
Yaw axis, °	19.9	(18.4, 21.4)	23.9	(22.4, 25.4)	0.00 ***
Dix–Hallpike Test (*n* = 191)	Right Side		Left Side		
Pitch axis, °	10.6	(9.4, 11.7)	11.3	(10.2, 12.4)	0.31
Yaw axis, °	7.0	(6.2, 7.8)	13.2	(12.2, 14.3)	0.00 ***
Comparison of the Supine Roll Test and the Dix–Hallpike Test					
Right Side (*n* = 189)	Supine Roll Test		Dix–Hallpike Test		
Pitch axis, °	8.2	(7.3, 9.0)	10.6	(9.4, 11.7)	0.00 **
Yaw axis, °	19.9	(18.4, 21.4)	7.0	(6.2, 7.8)	0.00 ***
Left Side (*n* = 187)	Supine Roll Test		Dix–Hallpike Test		
Pitch axis, °	7.4	(6.6, 8.3)	11.3	(10.2, 12.4)	0.00 ***
Yaw axis, °	23.9	(22.4, 25.4)	13.2	(12.2, 14.3)	0.00 ***

CI: confidence interval. All *p*-values are obtained from paired *t*-tests. A *p*-value is considered with a higher level of significance: *p* < 0.001 (**) and *p* < 0.0001 (***). Please note that the yaw axis head angles of the left sides had a significant higher absolute difference than the right sides in the same diagnostic test. When comparing the same axes of the same laterality between the Supine Roll Test and the Dix–Hallpike test, all measures are significantly different. The Supine Roll Test displays a bigger difference than the Dix–Hallpike test for the head angles on the yaw axis, and the Dix–Hallpike test displays a bigger difference than the Supine Roll Test for the head angles on the pitch axis.

**Table 4:** Head positioning accuracy in manual BPPV diagnostics: participant-related limitations (*n* = 198).

	Target Head Angle	Absolute Difference Between Target and Obtained Head Angles							
		Participants Without Reported Limitations (*n* = 159)		Participants with Reported Limitations During Manual Diagnostics (*n* = 39) ^a^					
				Participant-Reported Limitations (*n* = 21)			Examiner-Reported Compromised Cooperation (*n* = 37)		
		Mean	95% CI	Mean	95% CI	*p*-Value	Mean	95% CI	*p*-Value
Supine Roll Test		(*n* = 153)		(*n* = 21)			(*n* = 37)		
Right Side									
Pitch axis, °	−60.0	7.8	(6.9, 8.7)	8.15	(5.5, 10.8)	0.99	9.7	(7.2, 12.1)	0.15
Yaw axis, °	90.0	18.3	(16.8, 19.8)	23.1	(17.7, 28.6)	0.14	26.9	(22.8, 31.0)	0.00 **
Left Side									
Pitch axis, °	−60.0	7.1	(6.2, 8.0)	6.9	(5.5, 8.3)	0.43	8.9	(7.1, 10.7)	0.09
Yaw axis, °	−90.0	21.8	(20.3, 23.3)	32.4	(27.4, 37.5)	0.00 **	32.7	(29.2, 36.3)	0.00 ***
Right Dix–Hallpike Test		(*n* = 158)		(*n* = 20			(*n* = 35)		
Pitch axis, °	−120.0	10.4	(9.1, 11.6)	12.6	(9.0, 16.1)	0.27	11.8	(9.1, 14.6)	0.32
Yaw axis, °	45.0	7.0	(6.1, 7.9)	5.6	(3.3, 7.9)	0.23	6.9	(4.6, 9.2)	0.92
Left Dix–Hallpike Test		(*n* = 157)		(*n* = 19)			(*n* = 34)		
Pitch axis, °	−120.0	11.1	(9.9, 12.3)	14.1	(9.4, 18.8)	0.19	12.0	(9.2, 14.9)	0.53
Yaw axis, °	−45.0	13.2	(12.0, 14.3)	15.7	(11.5, 19.9)	0.12	13.9	(11.1, 16.7)	0.57

CI: confidence interval. ^a^ A total of 19 participants had both self- and examiner-reported compromised cooperation, 2 participants had only self-reported limitations, and 18 participants had only examiner-reported compromised cooperation. *p*-values were obtained with an unpaired *t*-test between the group with no reported limitations and each group with reported limitations. To control for Type I errors, all significant differences were Bonferroni-corrected. A *p*-value is considered with a higher level of significance if *p* < 0.001 (**) and *p* < 0.0001 (***). Please note that the absolute differences between the target and obtained head angles were not equal for both groups that experienced limitations during manual diagnostics compared to the group with no limitations for most of the head angles. This suggests that the variance in head angulation is poorly associated with the presence of participant-reported limitations and examiner-reported compromised cooperation.

**Table 5:** Head positioning accuracy in manual BPPV diagnostics: influence of participant-reported limitations (*n* = 198).

	Participant-Reported Limitations (*n* = 21)			
	β	95% CI	*p*-Value	Adjusted R^2^
Supine Roll Test (*n* = 192)				
Right Side				
Pitch axis, °	−0.8	(−3.8, 2.2)	0.60	0.01
Yaw axis, °	0.0	(−4.6, 4.6)	0.99	0.17
Left Side				
Pitch axis, °	−1.2	(−3.9, 1.5)	0.38	0.01
Yaw axis, °	7.2	(2.6, 11.8)	0.00 *	0.15
Right Dix–Hallpike Test (*n* = 195)				
Pitch axis, °	2.2	(−1.8, 6.3)	0.27	0.00
Yaw axis, °	−1.5	(−4.1, 1.0)	0.24	0.00
Left Dix–Hallpike Test (*n* = 193)				
Pitch axis, °	2.8	(−1.7, 7.3)	0.221	0.01
Yaw axis, °	2.1	(−1.5, 5.7)	0.25	0.01

β: β-coefficient; CI: confidence interval. The effect of the participant-reported limitations was analyzed using a multiple regression analysis adjusted for age. A *p*-value is considered significant if *p* < 0.05 (*). Please note that all adjusted R^2^s are low, which suggests that the model has little explanatory power for the intra-rater accuracy in the head angles.

**Table 6:** Angular velocity and movement duration in manual BPPV diagnostics (*n* = 198).

	Mean Angular Velocity (Degrees/Second)			Peak Angular Velocity (Degrees/Second)			Movement Duration (Seconds)		
	Mean [Range]	SD	COV	Mean [Range]	SD	COV	Mean [Range]	SD	COV
Supine Roll Test (*n* = 192)									
Supine to right side	34.0 [11.4, 68.0]	±9.7	0.29	144.4 [57.8, 289.0]	±42.9	0.30	1.39 [1.2, 2.7]	±0.2	0.14
Right to left side	67.9 [32.7, 119.7]	±13.8	0.20	217.6 [87.5, 353.6]	±59.5	0.27	1.80 [1.3, 3.5]	±0.3	0.17
Right Dix–Hallpike Test (*n* = 195)									
Upright to supine position	46.3 [21.4, 90.8]	±9.7	0.21	133.4 [37.9, 257.3]	±37.6	0.21	2.08 [1.1,4.1]	±0.4	0.19
Left Dix–Hallpike Test (*n* = 193)									
Upright to supine position	45.8 [14.7, 93.8]	±10.3	0.22	121.9 [41.9, 206.9]	±30.8	0.25	2.12 [1.0, 6.7]	±0.5	0.24

SD: standard deviation, COV: coefficient of variance. Please note the large range in the mean and the peak angular velocity, as well as the total duration of all movements. This shows the very high variability in and very low reproducibility of both velocities between individual test positions, as well as the total movement duration for manual diagnostic testing.

## Data Availability

The raw data supporting the conclusions of this article will be made available by the authors on request.

## Supplementary Materials

The following supporting information can be downloaded at https://www.mdpi.com/article/10.3390/jcm14020434/s1. Table S1: Difference between the target and obtained head angles by study period; Table S2: Comparison of included and excluded participants; Figure S1: Inertial measurement unit sensor data from the Supine Roll Test; Figure S2: Inertial measurement unit sensor data from the right side in the Dix–Hallpike test; Figure S3: Inertial measurement unit sensor data from the left side in the Dix–Hallpike test.

## Abbreviations

6DOF	Six degrees of freedom
BPPV	Benign paroxysmal positional vertigo
COV	Coefficient of variance
DHT	Dix–Hallpike test
ENT	Otorhinolaryngologist
IMU	Inertial measurement unit
SCC	Semicircular canal
SD	Standard deviation
SRT	Supine Roll Test
VNG	Videonystagmography
