# Supplementary material for: The Intra-Examiner Variability in and Accuracy of Traditional Manual Diagnostics of Benign Paroxysmal Positional Vertigo: A Prospective Observational Cohort Study
Source: J Clin Med. 2025 Jan 11;14(2):434. doi: 10.3390/jcm14020434 (PMC11766315; doi:10.3390/jcm14020434)
Supplement: Supplementary file 1 [file jcm-14-00434-s001.zip › jcm-3367486-supplementary.pdf]

Supplementary Material

Table S1. Difference Between the Target and the Obtained Head Angles by Study Period (n=198).

**Target Head Angle**		**First Study Period (n=99)**		**Second Study Period (n=99)**		
		Mean	*95% CI*	*Mean*	*95% CI*	*p-value*
**Supine Roll Test**		**(n=95)**		**(n=97)**		
Right side						
Pitch axis, °	-60.0	4.5	(2.3, 6.6)	4.4	(2.8, 6.0)	0.95
Yaw axis, °	90.0	21.4	(19.0, 23.8)	18.0	(16.0, 20.1)	0.04*
Left side						
Pitch axis, °	-60.0	6.6	(4.8, 8.5)	0.3	(-1.1, 1.7)	0.00**
Yaw axis, °	-90.0	-26.5	(-28.7, -24.2)	-21.2	(-23.2, -19.3)	0.00*
**Right Dix-Hallpike Test**		**(n=98)**		**(n=97)**		
Pitch axis, °	-120.0	-5.7	(-8.1, -3.4)	-9.8	(-11.7, -7.9)	0.01*
Yaw axis, °	45.0	-4.9	(-6.8, -3.0)	0.0	(-1.5, 1.5)	0.00**
**Left Dix-Hallpike Test**		**(n=95)**		**(n=98)**		
Pitch axis, °	-120.0	-5.4	(-7.7, -3.1)	-11.9	(-13.6, -10.2)	0.00*
Yaw axis, °	-45.0	-8.3	(-10.4, -6.2)	-15.1	(-16.6, -13.6)	0.00*

All p-values were obtained with unpaired t-tests of the means between the groups defined by study the first and second study periods (in case of unequal variance, Welch's t-test was used). A p-value is considered significant if $p<0.05$ (*). A higher level of significance is marked in the following manner: $p<0.001$ (**) and $p<0.0001$ (***). Please note that there was a significant difference between the two study periods for every variable except one. However, the direction of the difference was no consistent pattern, suggesting that the direction of the difference was random (no learning curve).

Table S2. Comparison of Included and Excluded Participants (n=279).

	**Included (n=198)**		**Excluded (n=81)**		***p-Value***
**Sex:**					
Female, n (%)	140	(70.7)	52	(64.2)	0.40
Age, years (mean, ± SD)	58.6	± 15.7	59.1	15.7	0.81

A p-value is considered significant when $p<0.05$. Please note that there was no significant difference between the included and excluded participants in terms of sex and age.

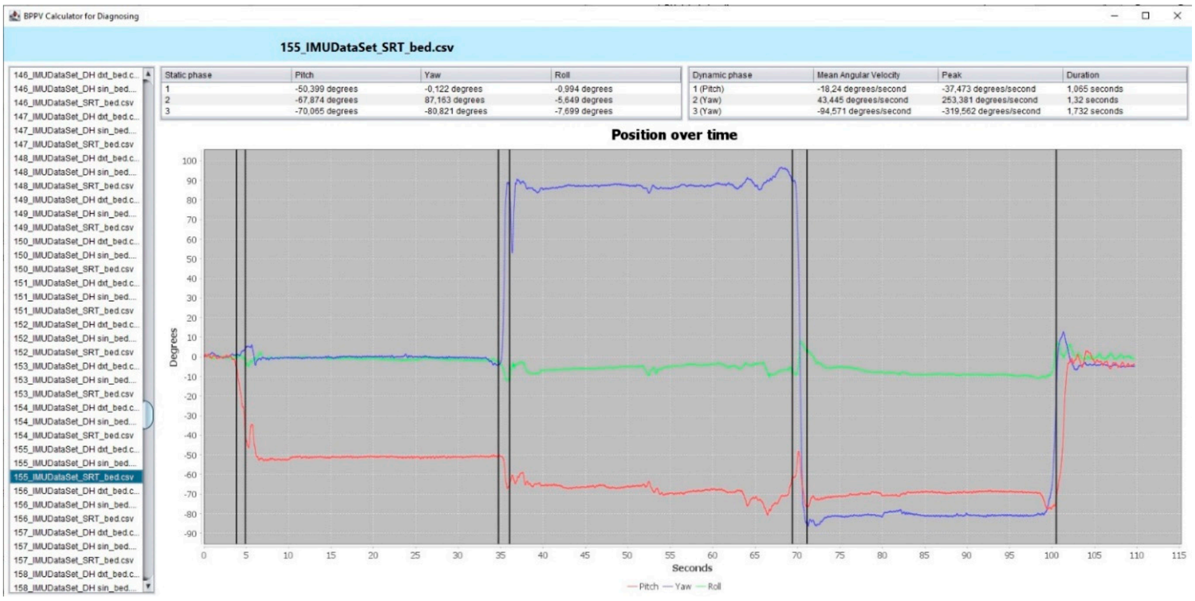

Figure S1. Inertial measurement unit (IMU) sensor data from the Supine Roll Test (SRT). A screenshot of the software that analyzed and visualized the raw data from the 6 degrees of freedom IMU sensor was recorded during the SRT. The software was programmed to separate (marked with black lines) the static phases (the supine position and the right and left SRT) and the dynamic phases. The software calculated the mean head angle for the pitch, yaw, and roll axes of the static phases. For the dynamic phases, the angular velocities (mean and peak) and the movement duration were calculated. .

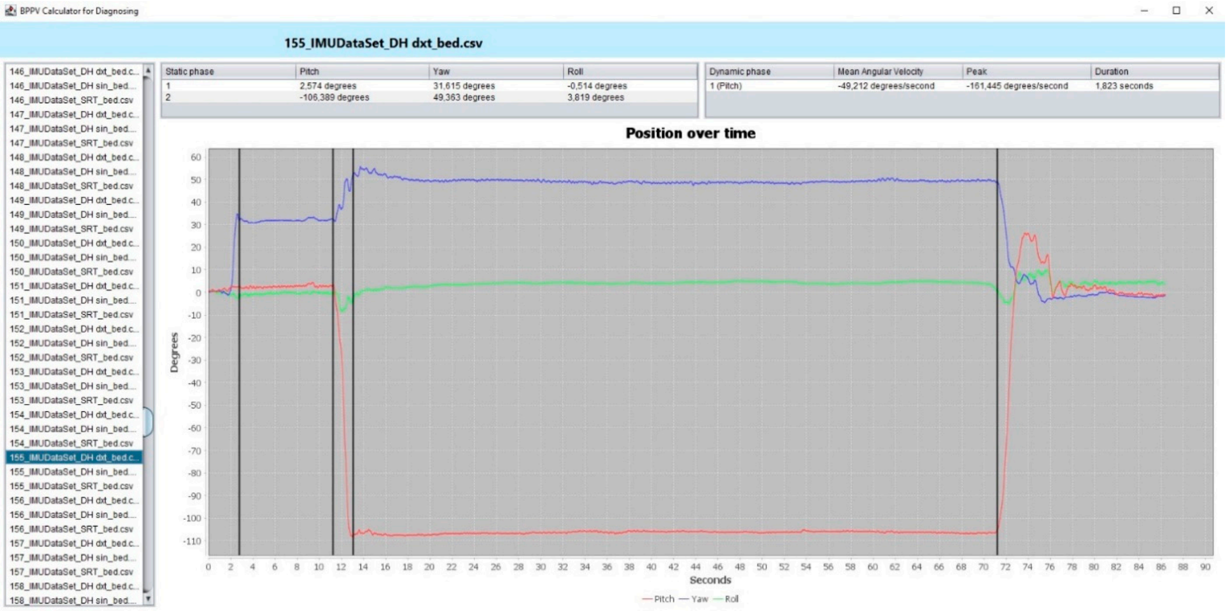

Figure S2. Inertial measurement unit (IMU) sensor from the right Dix-Hallpike Test (DHT). A screenshot of the software that analyzed and visualized the raw data from the 6 degrees of freedom IMU sensor was recorded during the right DHT. The software was programmed to separate (marked with black lines) the static phases

(upright position and the supine position with neck extension) and the dynamic phase. The software calculated the mean head angle for the pitch, yaw, and roll axes of the static phases. For the dynamic phase, the angular velocities (mean and peak) and the movement duration were calculated.

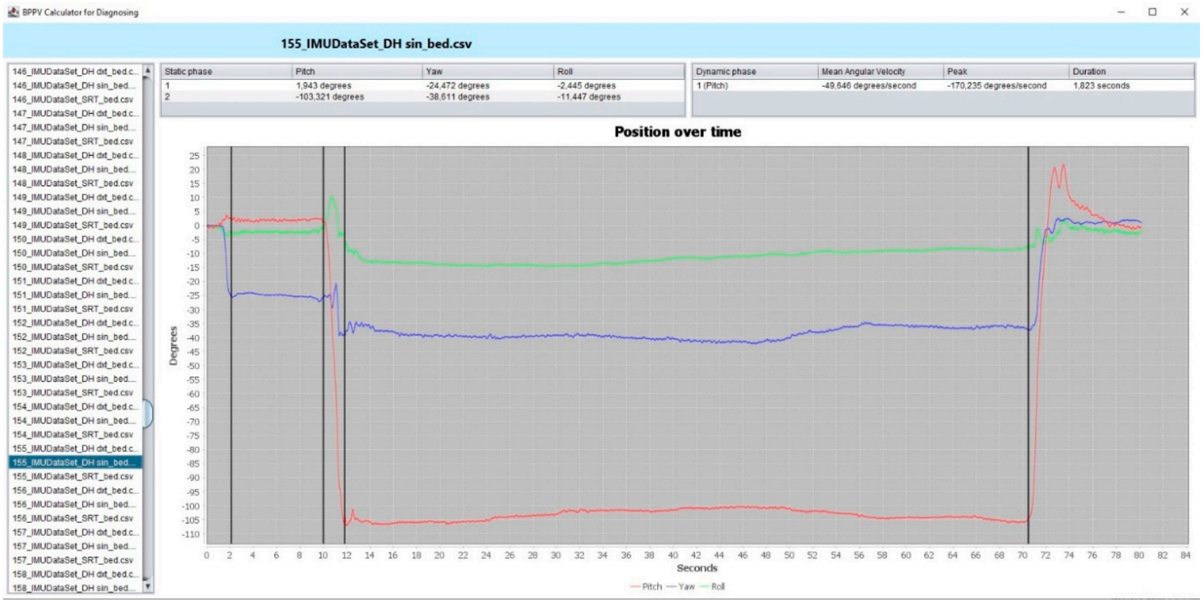

Figure S3. Inertial measurement unit (IMU) sensor from the left Dix-Hallpike Test (DHT). A screenshot of the software that analyzed and visualized the raw data from the 6 degrees of freedom IMU sensor was recorded during the left DHT. The software was programmed to separate (marked with black lines) the static phases (upright position and the supine position with neck extension) and the dynamic phase. The software calculated the mean head angle for the pitch, yaw, and roll axes of the static phases. For the dynamic phase, the angular velocities (mean and peak) and the movement duration were calculated.
